# Developing a novel parameter-free optimization framework for flood routing

**DOI:** 10.1038/s41598-021-95721-0

**Published:** 2021-08-10

**Authors:** Omid Bozorg-Haddad, Parisa Sarzaeim, Hugo A. Loáiciga

**Affiliations:** 1 Department of Irrigation and Reclamation Engineering, Faculty of Agricultural Engineering and Technology, College of Agriculture and Natural Resources, University of Tehran, Karaj, Tehran 31587-77871 Iran; 2 Department of Geography, University of California, Santa Barbara, CA 93016-4060 USA

**Keywords:** Climate sciences, Hydrology, Natural hazards

## Abstract

The Muskingum model is a popular hydrologic flood routing technique; however, the accurate estimation of model parameters challenges the effective, precise, and rapid-response operation of flood routing. Evolutionary and metaheuristic optimization algorithms (EMOAs) are well suited for parameter estimation task associated with a wide range of complex models including the nonlinear Muskingum model. However, more proficient frameworks requiring less computational effort are substantially advantageous. Among the EMOAs teaching–learning-based optimization (TLBO) is a relatively new, parameter-free, and efficient metaheuristic optimization algorithm, inspired by the teacher-student interactions in a classroom to upgrade the overall knowledge of a topic through a teaching–learning procedure. The novelty of this study originates from (1) coupling TLBO and the nonlinear Muskingum routing model to estimate the Muskingum parameters by outflow predictability enhancement, and (2) evaluating a parameter-free algorithm’s functionality and accuracy involving complex Muskingum model’s parameter determination. TLBO, unlike previous EMOAs linked to the Muskingum model, is free of algorithmic parameters which makes it ideal for prediction without optimizing EMOAs parameters. The hypothesis herein entertained is that TLBO is effective in estimating the nonlinear Muskingum parameters efficiently and accurately. This hypothesis is evaluated with two popular benchmark examples, the Wilson and Wye River case studies. The results show the excellent performance of the “TLBO-Muskingum” for estimating accurately the Muskingum parameters based on the Nash–Sutcliffe Efficiency (*NSE*) to evaluate the TLBO’s predictive skill using benchmark problems. The NSE index is calculated 0.99 and 0.94 for the Wilson and Wye River benchmarks, respectively.

## Introduction

The complex nature of flood events may challenge the straightforward, rapid, and accurate hydrological analysis. On the other hand, accurate and rapid-response modeling and simulation are necessary for flood routing in terms of minimization of damages and economic costs^1^. Hydrograph routing is a technique employed to simulate the changes in the shape of a flood hydrograph as inflow charges through a river channel or a reservoir^2,3^. These methods can be generally classified into two categories: hydraulic and hydrologic models^4^. The former models, such as Hydrologic Engineering Center River Analysis System (HEC-RAS) and MIKE11, are relatively complex compared to hydrologic routing models due to their dependency on solving the continuity, the energy, and/or the momentum equations^5,6^. What’s more, hydraulic routing models require detailed data and information river geometry and Manning's roughness coefficient, whose determination is time consuming and expensive and challengeable for calibration purposes^4^. On the other hand, hydrologic routing models, such as the Muskingum method, are more popular because of their simplicity, even though hydraulic routing methods are more accurate and have a more physically-based foundation. Yet, the robust calibration of hydrologic models has improved their accuracy to acceptable levels^7^. The three-parameter nonlinear Muskingum model features storage-time parameter ($K$), dimensionless river reach weighting factor ($\chi$), and dimensionless nonlinear flood wave (*m*) parameters, which must be estimated by traditional mathematical techniques or evolutionary and metaheuristic optimization algorithms (EMOAs)^8^.

Among the mathematical techniques one can cite the segmented least-squares method (S-LSM) by Gill^9^, univariate least squares (ULS) by Heggen^10^, least squares (LS) by Aldama^11^, the nonlinear least-squares method (N-LSM) by Yoon and Padmanabhan^12^, feasible sequential quadratic programming^13^, the Lagrange multiplier (LM) method by Das^14^, the Broyden–Fletcher–Goldfarb–Shannon (BFGS) technique by Geem^15^, and the Newton-type trust region algorithm (NTRA) by Sheng et al.^16^, among others. These mentioned techniques are relatively straightforward; yet, they are computationally burdensome and are commonly trapped into local optima. Moreover, the performance of the mathematical techniques is highly dependent on the quality of an initial search point, which means that the search for a solution is likely to converge to a local optimum, instead of a global optimum if the initial search point is not selected near the unknown global solution^17^.

Among the EMOAs are the genetic algorithm (GA) by Mohan^18^, harmony search (HS) by Kim et al.^17^ and by Geem et al.^19^, the ant colony algorithm (ACA) by Zhan and Xu^20^, the gray-encoded accelerating genetic algorithm (GEAGA) by Chen and Yang^21^, particle swarm optimization (PSO) by Chu and Chang^22^, the immune clonal selection algorithm (ICSA) by Luo and Xie^23^, the parameter setting free harmony search (PSF-HS) algorithm by Geem^8^, the imperialist competitive algorithm (ICA) by Tahershamsi and Sheikholeslami^24^, multi-objective particle swarm optimization (MOPSO) by Azadnia and Zahraie^25^, differential evolution (DE) by Xu et al.^26^, a combination of the simulated annealing (SA) algorithm and hybrid harmony search algorithm (HHSA) by Karahan et al.^27^, modified honey-bee mating optimization (MHBMO) algorithm by Niazkar and Afzali^28^, the backtracking search algorithm (BSA) by Yuan et al.^29^, Weed Optimization Algorithm (WOA) for extended nonlinear Muskingum model by Hamedi et al.^30^, PSO for a new form of Muskingum (four-parameter Muskingum model proposed by Easa^31^) by Moghaddam et al.^32^, modified PSO by Norouzi and Bazargan^33^, hybrid modified honey-bee mating (HMHBM) algorithm by Niazkar and Afzali^34^, bat algorithm (BA) by Farzin et al.^35^, wolf pack algorithm (WPA) by Bai et al.^36^, shark algorithm (SA) by Farahani et al.^37^. These and other algorithms have been previously applied to estimate the three parameters of the nonlinear form of the Muskingum parameters ($K$, $\chi$, and *m*). These EMOAs achieve an acceptable accuracy in the estimation of Muskingum parameters that are near global optima; yet, they must be pre-calibrated to assure their computational efficiency and accuracy. Sarzaeim et al.^38^ reported that all EMOAs involve controlling parameters including (1) general parameters, and (2) specific parameters. The group (1) parameters are required for all optimization algorithms (e.g., population size and number of iterations), whereas the group (2) parameters are specified by the optimization algorithms. In contrast, the relatively new teaching–learning-based optimization (TLBO) proposed by Rao et al.^39^ is a parameter-less method, i.e., TLBO does not involve controlling parameters. For purpose of illustration in comparison with other well-known EMOAs the GA features at least two specific parameters (the mutation and cross-over rates), and PSO involves at least three algorithmic parameters (learning factors, the variation of weight, and maximum velocity parameters)^39^. The recently-developed Cat Swarm Optimization (CSO) algorithm features four algorithmic parameters (seeking memory pool, seeking the range of selected dimension, counts of dimension to change, and self-position consideration)^40^. The Flower Pollination Algorithm (FPA) features four specific control parameters (the size of initial population, the scale factor for controlling step size, the levy distribution parameter, and the switch probability)^41^. Therefore, the calibration of the three-parameter nonlinear Muskingum model with each of the above EMOAs turns into a calibration of two sets of parameters, three for the routing model and those specific to the optimizing algorithm. The important advantage that distinguishes a parameter-free algorithm from other optimization algorithms stems from the fact that the output solutions of EMOAs are highly sensitive to the values of specific algorithmic parameters. Garousi-Nejad^42^ discussed how the quality performance of EMOAs are highly dependent on the tuning of algorithmic parameters. The main issue is that the optimization search may be stopped at local optima rather than achieving convergence to the global, best, solution. Optimization performance is highly sensitive to the specific algorithmic parameters pre-calibration, and sensitivity analysis is necessary to assign suitable values of the algorithmic parameters^5^. Moreover, Okkan and Kirdemir^43^ demonstrated less computational effort and faster convergence for optimization algorithms like PSO by developing modified algorithms with fewer control parameters. The cited studies seek to apply EMOAs with minimum controlling parameters.

This study proposes the TLBO algorithm coupled with nonlinear Muskingum routing to estimate the parameters of the flood routing model to overcome the limitations of the search techniques and the calibration of evolutionary algorithmic parameters. TLBO is a metaheuristic search algorithm with a significant merit that distinguishes it from other cited optimization algorithms because it does not involve algorithmic parameters^39^. In other words, its application does not require a pre-calibration process which leads to faster and more efficient estimation of Muskingum’s parameters. Proper calibration of the algorithmic parameters implies time-consuming computations and effort^44^. The novelty of this study is the application of a parameter-free TLBO, which constitutes a significant advantage in estimating the three parameters of the nonlinear Muskingum model. This work evaluates the “TLBO-Muskingum” framework’s performance The successful TLBO application in this work may encourage its use in other hydrologic problems.

**Figure 1:**
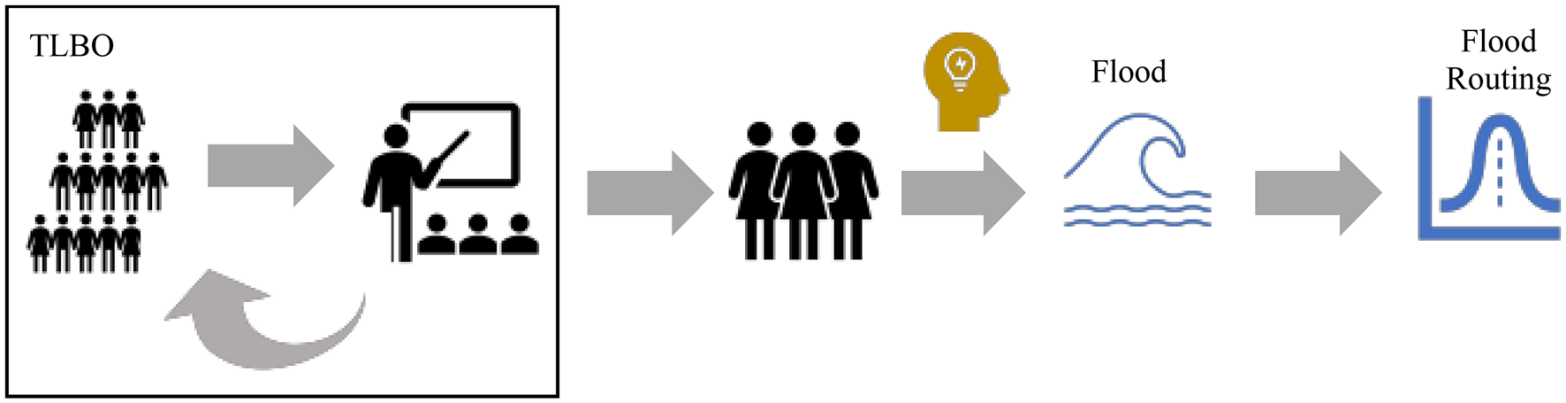
Graphical abstract.

This study couples the parameter-free TLBO and the nonlinear Muskingum model to estimate the model’s parameters by optimizing outflow predictions (Fig. 1). The hypothesis herein entertained is that “TLBO-Muskingum” can accurately predict outflow with less computational effort because it does not involve algorithm calibration. The TLBO’s performance is assessed by applying it to two well-known flood routing benchmark problems, relying on the Nash–Sutcliffe Efficiency (*NSE*) index as a hydrological performance criterion to evaluate the TLBO’s accuracy in outflow prediction.

## Methods

This study is structured as follow: the nonlinear Muskingum model is briefly presented in “The nonlinear Muskingum flood-routing model”. The TLBO algorithm, its functionality, and flowchart are described in “The teaching-learning-based optimization (TLBO) algorithm”. in detail. Next, the “TLBO-Muskingum” framework is introduced in “Linking TLBO to the Muskingum model”. The results from two flood routing benchmarks are discussed in “Results and discussion”, and the concluding remarks are presented in “Concluding remarks”.

### The nonlinear Muskingum flood-routing model

The relation between stream flow and reach storage is nonlinear; therefore, the original linear form of the Muskingum flood-routing model has been superseded by the following continuity and nonlinear Muskingum model, respectively^8,9,45^:

1 $$\frac{{dS_{t} }}{dt} = I_{t} - O_{t} ,$$

2 $$S_{t} = K[\chi I_{t} + (1 - \chi )O_{t} ]^{m} ,$$

where $S_{t}$, $I$, and $O_{t}$ denote the channel storage (with dimension of *L*^3^) of a river reach, rate of inflow with dimension of *L*^3^/*T* to a river reach, and rate of outflow (with dimension of *L*^3^/*T*) to a river reach, respectively, at time *t*; $K$, $\chi$, and *m* = storage-time constant parameter, dimensionless weighting factor, and dimensionless parameter related to the nonlinearity of the flood wave, respectively. The following Eq. (3) is derived from Eq. (2):

3 $$O_{t} = \frac{{(S_{t} /K)^{1/m} - \chi I_{t} }}{1 - \chi }.$$

Substituting Eq. (3) in Eq. (1) and taking the derivate of $S_{t}$ with respect to time produces:

4 $$\frac{{dS_{t} }}{dt} = I_{t} - \frac{{(S_{t} /K)^{1/m} - \chi I_{t} }}{1 - \chi } = \frac{{I_{t} - (S_{t} /K)^{1/m} }}{1 - \chi }.$$

Equation (4) is an ordinary first-order, nonlinear (when $m\ne 1)$, differential equation that does not have an analytical solution. Instead, Eq. (4) is routinely solved numerically. The following conditions are applied in the numerical solution of equation: (1) the inflow hydrograph $I_{t}$ is known, and (2) the initial inflow equals the initial outflow $O_{1} ( = I_{1} )$ Assumption (2) and Eq. (2) imply that $S_{1} ( = KO_{1}^{m} )$. Given values of the Muskingum parameters ($K$,$\chi$, and *m*) and applying the numerical discretization of the time derivative in Eq. (4) generates a recursive equation for reach storage $S_{t + 1}$ written in Eq. (5):

5 $$S_{t + 1} = S_{t} + \Delta t\left( {\frac{{\Delta S_{t} }}{\Delta t}} \right)\quad t = 1,2,3, \ldots$$

in which Δ*t* denotes the time step of hydrograph simulation. Therefore, the outflow $O_{t}$ is calculated with Eq. (6) as follows:

6 $$O_{t + 1} = \frac{{(S_{t + 1} /K)^{1/m} - \chi I_{t + 1} }}{1 - \chi }\quad t = 1,2,3, \ldots$$

The values of the parameters $K$,$\chi$, and *m* must be calibrated to achieve accurate outflow predictions with Eq. (6). Parameter calibration and hydraulic prediction can be achieved efficiently and accurately with EMOAs. The next section describes TLBO for the estimation of the Muskingum parameters with this parameter-free metaheuristic optimization algorithm in detail.

### The teaching–learning-based optimization (TLBO) algorithm

Teaching–learning-based optimization (TLBO) is a population-based, meta-heuristic, optimization algorithm inspired by the swarm intelligence of a population seeking to change from a current situation to an optimal situation by overall knowledge improvement (i.e. grades) of students in a classroom^39^. The peculiar feature of TLBO is that it does not require algorithmic parameters for its implementation other than general parameters ubiquitous to all evolutionary optimization algorithms such as population size and the number of iterations. Recall the GA features crossover and mutation rates, whose values affect the optimization performance and the accuracy of results^38^.

TLBO starts searching for the optimal solution of a well-posed problem with an initial population whose members’ scores are the values of the decision variables, such as grades earned by students. TLBO strives to improve the population’s quality by means of a “Teacher Phase” and a “Learner Phase” to achieve a solution that is very near to the globally optimal solution. In the following the “Teacher Phase” and “Learner Phase” are discussed.

In a classroom the teacher has the highest-level knowledge of a particular topic and endeavors to teach the students to advance their overall knowledge, and, consequently, raise their individual and average grades in exams. In the “Teacher Phase” the person with the best grade is considered as a teacher making efforts to transfer knowledge to the other learners (i.e. students) in a class. *I* Suppose there are *n* students in a class and that they have an average grade ${M}_{i}$ in exam *i*. The most successful student with the best grade ${X}_{T,i}$ in exam *i* among the *n* students plays the teacher’s role. The difference between the teacher’s level and the average level of knowledge (${Diff}_{i}$) in exam *i* is expressed as follows:

7 $$Diff_{i} = r_{i} \left( {X_{T,i} - T_{F} \times M_{i} } \right),$$

8 $$T_{F} = round \left[ {1 + rand \left( {0,1} \right)\left\{ {1,2} \right\}} \right],$$

in which, ${r}_{i}=$ random number in $\left[\mathrm{0,1}\right]$, and ${T}_{F}=$ a random number that accounts for the teacher factor that depends on teaching quality, and equals either 1 or 2.

By teaching and transferring knowledge to students their new, improved, grades in the next exam are defined by Eq. (9):

9 $$X^{\prime}_{j,i} = X_{j,i} + Diff_{i} ,$$

where, $X^{\prime}_{j,i}$ and $X_{j,i} =$ new and old grades of student *j* in exam *i*, respectively.$X_{j,i}$ is transferred to the “Learner Phase” if the old grade is better than the new one, otherwise $X^{\prime}_{j,i}$ is transferred.

In the “Learner Phase” the top students help their peers to improve their knowledge through, for example, team work in assignments, leading to new grade improvement ($X^{\prime\prime}$). This helping interaction between two students *A* and *B* in each exam *i* is defined as follow:

10 $$X_{A,i}^{^{\prime\prime}} = \left\{ {\begin{array}{*{20}c} {X^{\prime}_{A,i} + r_{i} (X^{\prime}_{A,i} - X^{\prime}_{B,i)} } & {if\,\,(X^{\prime}_{A,i} > X^{\prime}_{B,i} )} \\ {X^{\prime}_{A,i} + r_{i} (X^{\prime}_{B,i} - X^{\prime}_{A,i)} } & {if\,\,(X^{\prime}_{B,i} > X^{\prime}_{A,i} ).} \\ \end{array} } \right.$$

This teacher-students interaction is the fundamental inspiration for TLBO, in which the number of students in the class is the algorithm population size and the number of exams is the number of iterations. The TLBO steps are defined as follow:

1. Define the population size, the number of iterations, and the objective function.
2. Randomly initialize the grades (${X}_{i,j}$) of *n* students (*j* = 1, 2, …, *n*) in exam *i* = 1.
3. Evaluate the objective function for *n* students in exam *i*.
4. Select the student with the best grade as teacher and calculate ${Diff}_{i}$ for exam *i*.
5. To calculate $X^{\prime}_{j,i}$ for *n* students in exam *i*.
6. Compare $X_{j,i}$ and $X^{\prime}_{j,i}$ and select the better one for transferring to the next step.
7. Select randomly each pair of students and calculate $X_{j,i}^{^{\prime\prime}}$.
8. Compare $X^{\prime}_{j,i}$ and $X_{j,i}^{^{\prime\prime}}$ and select the better one for transferring to the next step.
9. Evaluate the objective function for all students, check whether the stop criterion is satisfied (the optimal solution is achieved), otherwise the algorithm will iterate from step (4).

A more in-depth description of TLBO can be found in Refs.^38,39,46^.

### Linking TLBO to the Muskingum model

**Figure 2:**
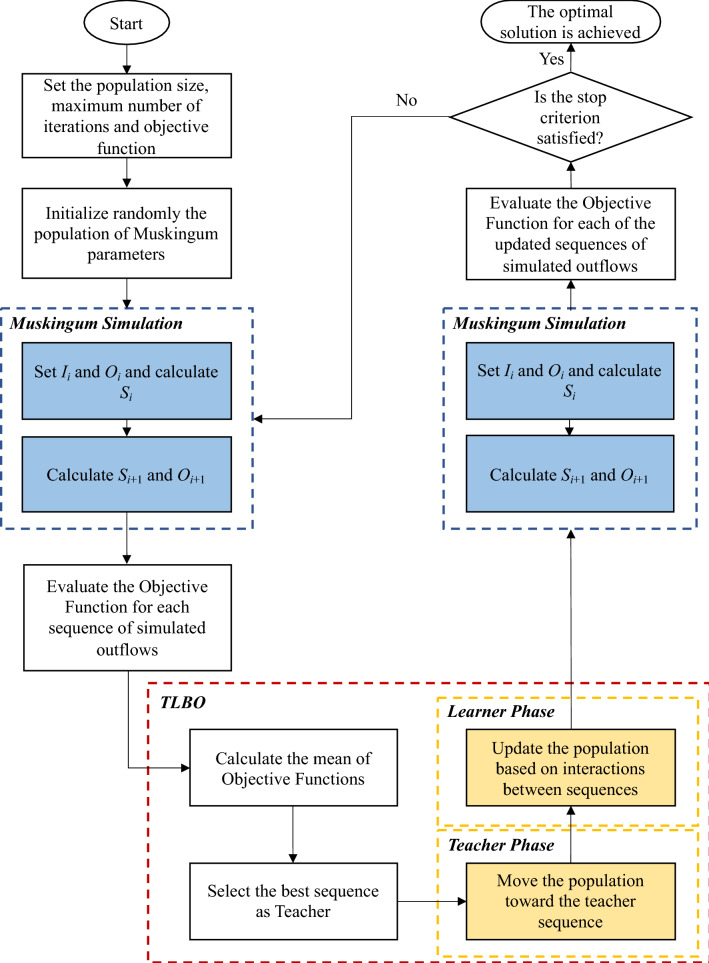
The flowchart of the “TLBO-Muskingum” model.

Figure 2 depicts the flowchart of the algorithmic “TLBO-Muskingum” method to estimate the Muskingum model parameters (i.e. $K$,$\chi$, and *m*). The algorithm starts by defining the population size (number of students), number of iterations (number of exams), and the objective function. Next, the initial population of Muskingum’s parameters is generated randomly. The flood hydrograph is then simulated with Eqs. (5) and (6). The values of the objective function for each sequence of scores earned by the students are calculated following the Muskingum simulation. The next step improves the current population of decision variables (i.e., the estimates of $K$,$\chi$, and *m*) by calculating the mean value of the objective function and selecting the best solution as the teacher of the population. Afterward, the population of parameters is updated in the teaching phase (i.e. moving the population toward the teachers’ sequence of simulated outflows) and the learning phase (i.e. updating the population based on the interaction between the students). In other words, the new population of parameters is generated with the modifier operator such that each student (or parameter estimate) starts moving towards the best solution in the population by means of the linear and random base equation (this is the Teacher Phase). Furthermore, the improvement of the population is guided by the interactions between students using a linear equation based on the difference between their positions (this is the so-called Learner Phase). The Muskingum simulation is repeated with the improved or updated population and the objective function is re-evaluated. The optimal solution is reported whenever the user-specified termination criterion is satisfied. Otherwise, the iterations involving improvement of the current population, Muskingum simulation, evaluation of objective functions, and assessment of the termination criterion proceed until convergence is achieved.

The population size is set equal to 100, the number of iterations is 500, and the objective function is expressed as below:

11 $$Min\,\,SSD = \mathop \sum \limits_{i = 1}^{T} \left( {O_{i} - \hat{O}_{i} } \right)^{2} \quad i = 1,2,3, \ldots$$

where, $SSD$ is the sum of the squared deviation between the observed and simulated outflows at time interval *t*, ${O}_{i}$ is the observed outflow at time interval *i*, and ${\widehat{O}}_{i}$ is the simulated outflow at time interval *i*.

## Results and discussion

The performance of the “TLBO-Muskingum” in estimating the three-parameters of nonlinear Muskingum model is evaluated with two well-known benchmark problems: (1) the Wilson cased study^47^, and (2) the River Wye in the United Kingdom^48^, both with one single peak flood event. The former one is a popular benchmark problem based on the data provided by Wilson (1974) and has been employed commonly in several studies^9,14,22,50–53^ to be linked to EMOAs for estimation of nonlinear Muskingum model’s parameters. The minor lateral nature of the flow makes this case study ideal for flood routing studies^48^.

The second case study is based on flood event of the River Wye in Dec 1960 in the United Kingdom^49^. The 69–75 km riverbed characteristics (without tributaries and small lateral inflow) make it ideal for flood-routing calibration purposes.

Along with objective function evaluation (*SSD*), the Nash–Sutcliffe Efficiency (*NSE*) is herein calculated for more precise evaluation of flood routing and optimization performance in hydrological point of view. The *NSE* is a normalized index of error variance^53^ that measures the predictive skill of hydrologic models. It takes value in the range of (− ∞, 1]. The closer *NSE* is to 1, the more accurately the hydrological model performs^54^. The *NSE* is defined as follows:

12 $$NSE = 1 - \frac{{\mathop \sum \nolimits_{i = 1}^{T} \left( {O_{i} - \hat{O}_{i} } \right)^{2} }}{{\mathop \sum \nolimits_{i = 1}^{T} \left( {O_{i} - \overline{O}} \right)^{2} }}\quad i = 1,2,3, \ldots$$

where *NSE* is the Nash–Sutcliffe Efficiency index, ${O}_{i}$ is the observed outflow at time interval *i*, ${\widehat{O}}_{i}$ is the simulated outflow at time interval *i*, and $\overline{O }$ is the average of observed outflow.

**Figure 3:**
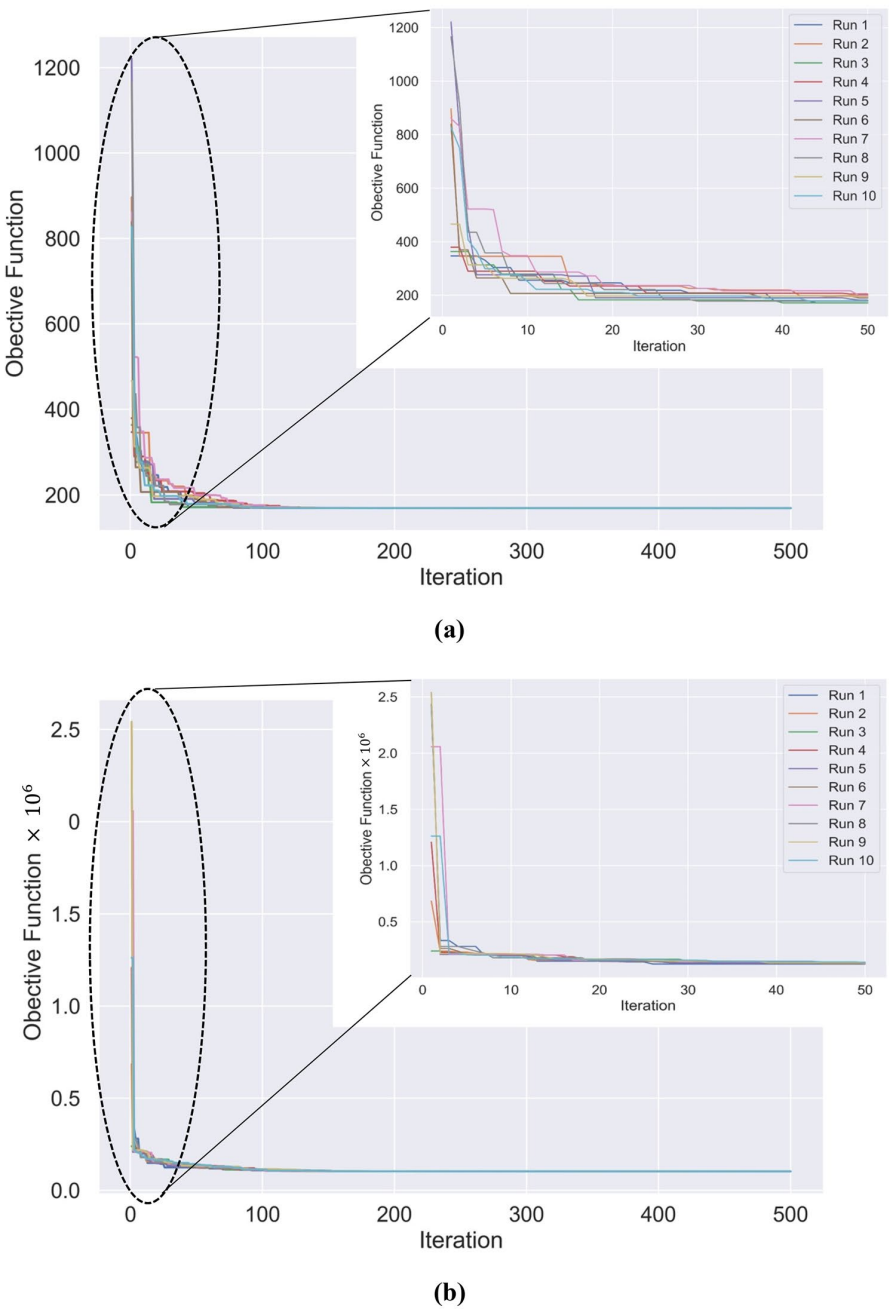
The objective function for 500 iterations in all 10 runs for (**a**) Wilson, and (**b**) Wye River benchmarks.

The “TLBO-Muskingum” has been implemented for 10 runs for each of the benchmark problems. The objective function values in all 500 iterations have been plotted in Fig. 3. It is shown in Fig. 3 that in both case studies the converges was achieved by iteration 500, the best objective function is 169.3 and 102,511.9 for the Wilson and Wye River benchmarks, respectively. For more precise insight, the zoom-in objective function values have been extracted up to iteration 50 in Fig. 3, which depicts the fast convergence of TLBO in reaching the optimal solution.

Figure 3 also illustrates how all 10 runs reached the same objective function, which results in negligible difference between the 10 runs for each benchmark problem. The calculated average, minimum (min), maximum (max), and standard deviation (*SD*) statistics of all the runs are listed in Tables 1 and 2 for the Wilson and Wye River benchmarks, respectively. These statistics are not significantly different at each time step and the *SD* values are small (the average standard deviation between the simulated outflows in all time steps for all 10 runs is $6\times {10}^{-6}$ and $7\times {10}^{-5}$ for the Wilson and Wye River examples, respectively). The small *SD* values stem from the high convergence capability of the TLBO in outflow simulations, which confirms the high reliability and robustness of “random-based” TLBO method in flow prediction. The average values of the simulated outflow were used for further analysis. It is important to notice that the results from the 10 runs were calculated without calibration of any algorithmic parameters. The observed and average simulated outflows timeseries calculated with the optimal values of the Muskingum parameters are listed in Tables 3 and 4 for the Wilson and Wye River case studies, respectively. The *SSD* as objective function from calculated outflows along with the *NSE* index as a standard hydrological performance metric are listed in Tables 3 and 4, as well. The calculated *SSD* is shown as function of the iteration number in Fig. 3. The *NSE* values clearly show how accurately the “TLBO-Muskingum” simulates the outflows, and consequently how the three parameters of Muskingum are estimated precisely. The *NSE* value for the Wilson case study is 0.99, demonstrating the excellent performance of the optimization process. The *NSE* equals 0.94 regarding the Wye River, also showing high algorithmic accuracy. Recall that the closer the *NSE* to 1, the more accurate the model prediction is.

**Table 1:** Outflows (m^3^/s) calculated in 10 runs of “TLBO-Muskingum” for the Wilson benchmark problem.

Time (h)	Run 1	Run 2	Run 3	Run 4	Run 5	Run 6	Run 7	Run 8	Run 9	Run 10	Average	Min	Max	*SD*
0	22.00	22.00	22.00	22.00	22.00	22.00	22.00	22.00	22.00	22.00	22.00	22.00	22.00	–
6	21.77	21.77	21.77	21.77	21.77	21.77	21.77	21.77	21.77	21.77	21.77	21.77	21.77	1.211E−07
12	19.91	19.91	19.91	19.91	19.91	19.91	19.91	19.91	19.91	19.91	19.91	19.91	19.91	1.825E−06
18	20.74	20.74	20.74	20.74	20.74	20.74	20.74	20.74	20.74	20.74	20.74	20.74	20.74	7.422E−06
24	32.41	32.41	32.41	32.41	32.41	32.41	32.41	32.41	32.41	32.41	32.41	32.41	32.41	7.48E−06
30	48.02	48.02	48.02	48.02	48.02	48.02	48.02	48.02	48.02	48.02	48.02	48.02	48.02	2.888E−06
36	60.43	60.43	60.43	60.43	60.43	60.43	60.43	60.43	60.43	60.43	60.43	60.43	60.43	4.517E−06
42	70.44	70.44	70.44	70.44	70.44	70.44	70.44	70.44	70.44	70.44	70.44	70.44	70.44	8.133E−06
48	78.12	78.12	78.12	78.12	78.12	78.12	78.12	78.12	78.12	78.12	78.12	78.12	78.12	1.052E−05
54	82.76	82.76	82.76	82.76	82.76	82.76	82.76	82.76	82.76	82.76	82.76	82.76	82.76	1.112E−05
60	84.57	84.57	84.57	84.57	84.57	84.57	84.57	84.57	84.57	84.57	84.57	84.57	84.57	1.013E−05
66	82.39	82.39	82.39	82.39	82.39	82.39	82.39	82.39	82.39	82.39	82.39	82.39	82.39	7.277E−06
72	78.55	78.55	78.55	78.55	78.55	78.55	78.55	78.55	78.55	78.55	78.55	78.55	78.55	4.455E−06
78	73.18	73.18	73.18	73.18	73.18	73.18	73.18	73.18	73.18	73.18	73.18	73.18	73.18	3.575E−06
84	65.84	65.84	65.84	65.84	65.84	65.84	65.84	65.84	65.84	65.84	65.84	65.84	65.84	5.8E−06
90	57.88	57.88	57.88	57.88	57.88	57.88	57.88	57.88	57.88	57.88	57.88	57.88	57.88	8.214E−06
96	48.70	48.70	48.70	48.70	48.70	48.70	48.70	48.70	48.70	48.70	48.70	48.70	48.70	9.694E−06
102	39.31	39.31	39.31	39.31	39.31	39.31	39.31	39.31	39.31	39.31	39.31	39.31	39.31	9.308E−06
108	30.89	30.89	30.89	30.89	30.89	30.89	30.89	30.89	30.89	30.89	30.89	30.89	30.89	7.257E−06
114	24.29	24.29	24.29	24.29	24.29	24.29	24.29	24.29	24.29	24.29	24.29	24.29	24.29	6.128E−06
120	20.03	20.03	20.03	20.03	20.03	20.03	20.03	20.03	20.03	20.03	20.03	20.03	20.03	5.204E−06
126	19.31	19.31	19.31	19.31	19.31	19.31	19.31	19.31	19.31	19.31	19.31	19.31	19.31	1.428E−06

**Table 2:** Outflows (m^3^/s) calculated in 10 runs of “TLBO-Muskingum” for the Wye River benchmark problem.

Time (h)	Run 1	Run 2	Run 3	Run 4	Run 5	Run 6	Run 7	Run 8	Run 9	Run 10	Average	Min	Max	*SD*
0	102.00	102.00	102.00	102.00	102.00	102.00	102.00	102.00	102.00	102.00	102.00	102.00	102.00	–
6	171.37	171.37	171.37	171.37	171.37	171.37	171.37	171.37	171.37	171.37	171.37	171.37445	171.37459	4.421E−05
12	129.60	129.60	129.60	129.61	129.60	129.60	129.60	129.60	129.60	129.60	129.60	129.60491	129.60504	3.521E−05
18	219.44	219.44	219.44	219.44	219.44	219.44	219.44	219.44	219.44	219.44	219.44	219.44392	219.44404	3.686E−05
24	189.89	189.89	189.89	189.89	189.89	189.89	189.89	189.89	189.89	189.89	189.89	189.88594	189.88596	3.827E−06
30	180.26	180.26	180.26	180.26	180.26	180.26	180.26	180.26	180.26	180.26	180.26	180.25707	180.2571	6.035E−06
36	197.97	197.97	197.97	197.97	197.97	197.97	197.97	197.97	197.97	197.97	197.97	197.97446	197.97451	1.255E−05
42	172.79	172.79	172.79	172.79	172.79	172.79	172.79	172.79	172.79	172.79	172.79	172.78686	172.7869	1.236E−05
48	155.64	155.64	155.64	155.64	155.64	155.64	155.64	155.64	155.64	155.64	155.64	155.63766	155.6377	1.225E−05
54	110.41	110.41	110.41	110.41	110.41	110.41	110.41	110.41	110.41	110.41	110.41	110.41004	110.41017	3.507E−05
60	148.17	148.17	148.17	148.17	148.17	148.17	148.17	148.17	148.17	148.17	148.17	148.16797	148.1681	3.558E−05
66	172.98	172.98	172.98	172.98	172.98	172.98	172.98	172.98	172.98	172.98	172.98	172.97914	172.97943	8.922E−05
72	251.59	251.59	251.59	251.59	251.59	251.59	251.59	251.59	251.59	251.59	251.59	251.58505	251.58558	0.0001552
78	302.30	302.30	302.30	302.30	302.30	302.30	302.30	302.30	302.30	302.30	302.30	302.2999	302.30091	0.0002829
84	480.43	480.43	480.43	480.43	480.43	480.43	480.43	480.43	480.43	480.43	480.43	480.42843	480.42946	0.0002876
90	764.70	764.70	764.70	764.70	764.70	764.70	764.70	764.70	764.70	764.70	764.70	764.70416	764.70439	7.868E−05
96	801.86	801.86	801.86	801.86	801.86	801.86	801.86	801.86	801.86	801.86	801.86	801.86123	801.86152	8.264E−05
102	741.45	741.45	741.45	741.45	741.45	741.45	741.45	741.45	741.45	741.45	741.45	741.45154	741.452	0.0001242
108	637.53	637.53	637.53	637.53	637.53	637.53	637.53	637.53	637.53	637.53	637.53	637.52539	637.52589	0.0001346
114	568.66	568.66	568.66	568.66	568.66	568.66	568.66	568.66	568.66	568.66	568.66	568.65752	568.65816	0.0001731
120	453.41	453.41	453.41	453.41	453.41	453.41	453.41	453.41	453.41	453.41	453.41	453.40569	453.40632	0.0001762
126	333.33	333.33	333.33	333.33	333.33	333.33	333.33	333.33	333.33	333.33	333.33	333.33322	333.33375	0.0001557
132	219.50	219.50	219.50	219.50	219.50	219.50	219.50	219.50	219.50	219.50	219.50	219.50007	219.50043	0.0001115
138	129.19	129.19	129.19	129.19	129.19	129.19	129.19	129.19	129.19	129.19	129.19	129.18819	129.18842	7.404E−05
144	112.42	112.42	112.42	112.42	112.42	112.42	112.42	112.42	112.42	112.42	112.42	112.41943	112.41949	1.761E−05
150	97.45	97.45	97.45	97.45	97.45	97.45	97.45	97.45	97.45	97.45	97.45	97.448267	97.448341	2.304E−05
156	90.45	90.45	90.45	90.45	90.45	90.45	90.45	90.45	90.45	90.45	90.45	90.446248	90.446295	1.457E−05
162	87.21	87.21	87.21	87.21	87.21	87.21	87.21	87.21	87.21	87.21	87.21	87.206914	87.206973	1.83E−05
168	77.42	77.42	77.42	77.42	77.42	77.42	77.42	77.42	77.42	77.42	77.42	77.424034	77.424084	1.435E−05
174	74.86	74.86	74.86	74.86	74.86	74.86	74.86	74.86	74.86	74.86	74.86	74.857992	74.858069	2.236E−05
180	71.03	71.03	71.03	71.03	71.03	71.03	71.03	71.03	71.03	71.03	71.03	71.025309	71.025419	3.153E−05
186	69.17	69.17	69.17	69.17	69.17	69.17	69.17	69.17	69.17	69.17	69.17	69.1709	69.171087	5.39E−05
192	64.23	64.23	64.23	64.23	64.23	64.23	64.23	64.23	64.23	64.23	64.23	64.231879	64.232212	9.517E−05
198	61.68	61.68	61.68	61.68	61.68	61.68	61.68	61.68	61.68	61.68	61.68	61.680446	61.681126	0.0001951

**Table 3:** Observed and average simulated outflow timeseries for the Wilson benchmark problem.

*I*	Time (h)	$O_{i} \,({\text{m}}^{3} /{\text{s}})$	$\hat{O}_{i} \,({\text{m}}^{3} /{\text{s}})$	$(O_{i} - \hat{O}_{i} )^{2}$	$(O_{i} - \overline{O})^{2}$
0	0	22.00	22.00	0.00	690.26
1	6	21.00	21.77	0.59	743.80
2	12	21.00	19.91	1.20	743.80
3	18	26.00	20.74	27.67	469.07
4	24	34.00	32.41	2.52	203.71
5	30	44.00	48.02	16.15	18.26
6	36	55.00	60.43	29.53	45.26
7	42	66.00	70.44	19.72	314.26
8	48	75.00	78.12	9.76	714.35
9	54	82.00	82.76	0.58	1137.53
10	60	85.00	84.57	0.18	1348.89
11	66	84.00	82.39	2.60	1276.44
12	72	80.00	78.55	2.09	1006.62
13	78	73.00	73.18	0.03	611.44
14	84	64.00	65.84	3.37	247.35
15	90	54.00	57.88	15.06	32.80
16	96	44.00	48.70	22.06	18.26
17	102	36.00	39.31	10.92	150.62
18	108	30.00	30.89	0.78	333.89
19	114	25.00	24.29	0.51	541.62
20	120	22.00	20.03	3.89	690.26
21	126	19.00	19.31	0.10	856.89
Sum	–	–	–	169.31	12,222.36
				*SSD* = 169.31	*NSE* = 0.99

**Table 4:** Observed and simulated outflows for the Wye River Benchmark problem.

*i*	Time (h)	$O_{i} \,({\text{m}}^{3} /{\text{s}})$	$\hat{O}_{i} \,({\text{m}}^{3} /{\text{s}})$	$(O_{i} - \hat{O}_{i} )^{2}$	$(O_{i} - \overline{O})^{2}$
0	0	102.00	102.00	0.00	26,110.76
1	6	140.00	171.37	984.36	15,274.05
2	12	169.00	129.60	1551.97	8946.93
3	18	190.00	219.44	866.95	5415.23
4	24	209.00	189.89	365.35	2979.88
5	30	218.00	180.26	1424.53	2078.29
6	36	210.00	197.97	144.61	2871.70
7	42	194.00	172.79	450.00	4842.52
8	48	172.00	155.64	267.73	8388.40
9	54	149.00	110.41	1489.18	13,130.46
10	60	136.00	148.17	148.06	16,278.76
11	66	228.00	172.98	3027.29	1266.52
12	72	303.00	251.59	2643.48	1553.29
13	78	366.00	302.30	4057.67	10,488.17
14	84	456.00	480.43	596.76	37,022.29
15	90	615.00	764.70	22,411.35	123,490.23
16	96	830.00	801.86	791.78	320,822.29
17	102	969.00	741.45	51,778.15	497,605.76
18	108	665.00	637.53	754.83	161,131.40
19	114	519.00	568.66	2465.92	65,235.17
20	120	444.00	453.41	88.48	32,548.40
21	126	321.00	333.33	152.12	3296.11
22	132	208.00	219.50	132.26	3090.05
23	138	176.00	129.19	2191.33	7671.70
24	144	148.00	112.42	1265.98	13,360.64
25	150	125.00	97.45	759.10	19,206.70
26	156	114.00	90.45	554.78	22,376.64
27	162	106.00	87.21	353.18	24,834.05
28	168	97.00	77.42	383.22	27,751.64
29	174	89.00	74.86	200.00	30,481.05
30	180	81.00	71.03	99.49	33,338.46
31	186	76.00	69.17	46.64	35,189.35
32	192	71.00	64.23	45.81	37,090.23
33	198	66.00	61.68	18.65	39,041.11
Sum	–	–	–	102,510.99	1,654,208.24
				*SSD* = 102,510.99	*NSE* = 0.94

**Figure 4:**
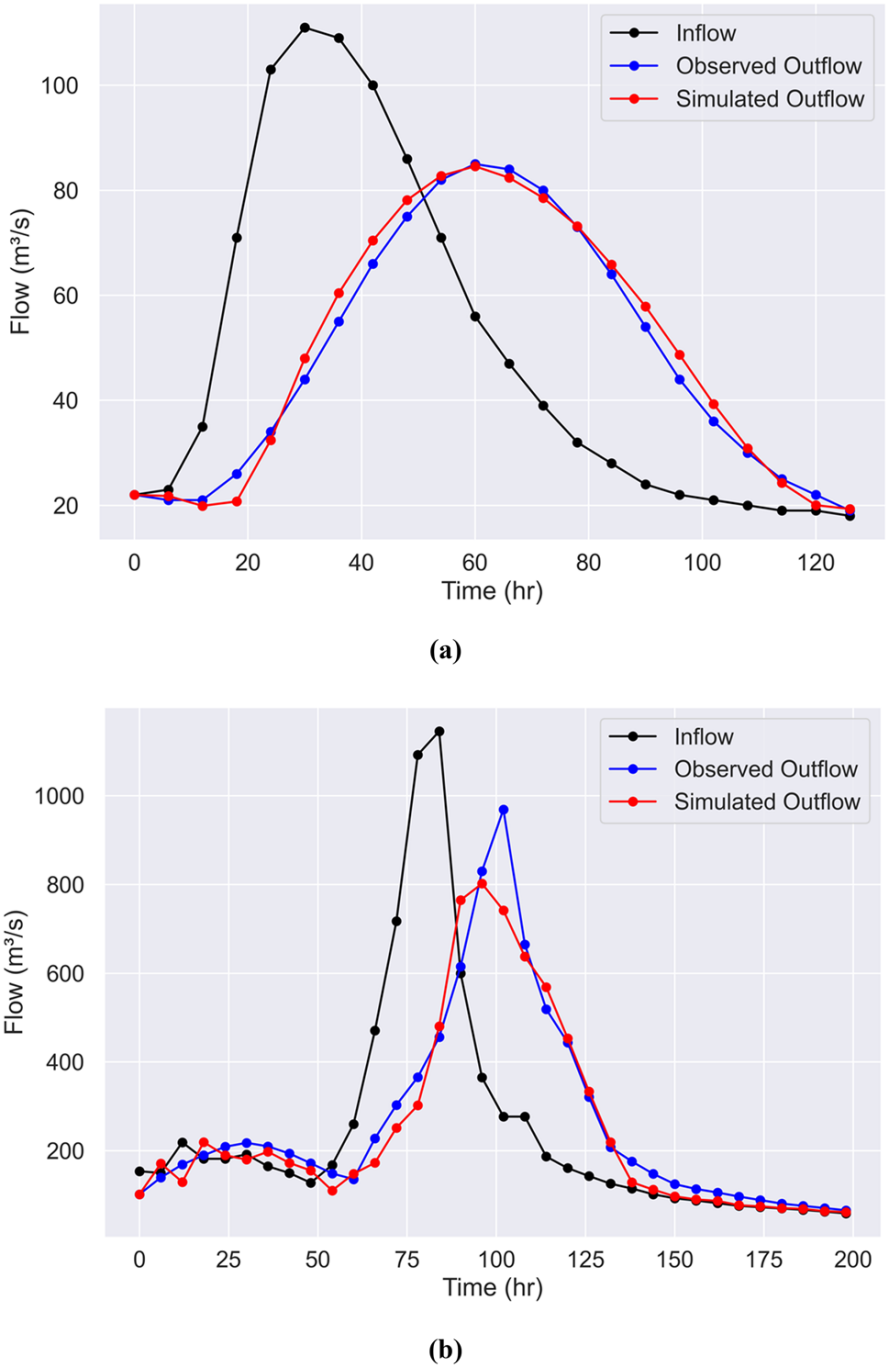
Inflow, observed, and average simulated outflow hydrographs in (**a**) Wilson, and (**b**) Wye River benchmarks.

**Figure 5:**
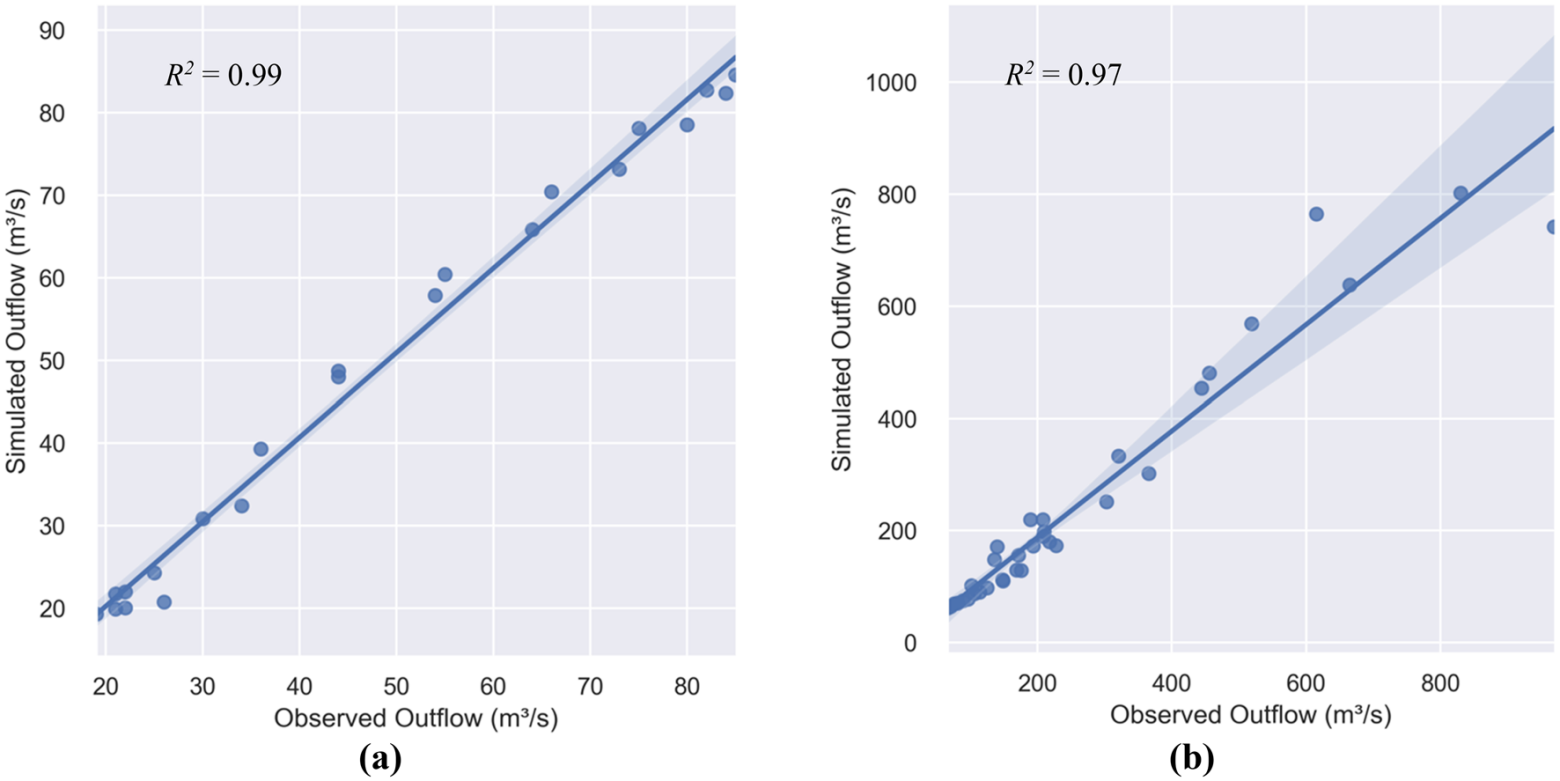
The scatter plots of the observed and simulated outflows corresponding to (**a**) the Wilson, and (**b**) the Wye River benchmarks.

Figure 4 depicts the timeseries of the observed and simulated hydrographs for case studies 1 and 2, along with the inflows. The simulated outflow hydrographs correspond to the average estimates of the Muskingum parameters. It is seen in Fig. 4 the overall good fit between observations and predictions. The accuracy of the “TLBO-Muskingum” algorithm’s predictions is relatively higher for the first benchmark problem (*NSE* = 0.99 and *R*^*2*^ = 0.99, see Table 5). The accuracy is nevertheless high in the second case study (*NSE* = 0.94 and *R*^*2*^ = 0.97, see Table 5), except for the peak outflow: which is underestimated. This is evident in Fig. 5 illustrating the scatterplots of the observed and simulated outflows. Clearly in the case of the Wilson example the accuracy is high for the entire range of the outflows (*R*^2^ = 0.99), while as discussed earlier, the predictive skill of the TLBO-Muskingum method for estimating lower outflows is superior to that associated with the peak outflows. The prediction of high flows may be improved by using a longer time series in the training phase of the Muskingum model. Furthermore, applying TLBO to the four-parameter Muskingum model may lead to better performance of the flood routing model with respect to peak flows, which may be addressed in future work.

**Table 5:** Muskingum model parameters with performance metrics and run time.

Case study	Muskingum model parameters			Performance metric		Run time (s)
	$K$	$\chi$	*m*	*R*^*2*^	*NSE*	
Wilson	0.0703	0.1895	2.1339	0.99	0.99	2.531
Wye River	0.0103	0.2221	2.1493	0.97	0.94	3.488

Table 5 lists the optimal values of the Muskingum’s parameters calculated by TLBO ($K$,$\chi$, and *m*), the *NSE,* and the average run time obtained with 10 runs. The average run time for the Wilson and Wye examples equal 2.531 and 3.488 s*,* respectively. This shows the rapid convergence of the TLBO-Muskingum method.

**Figure 6:**
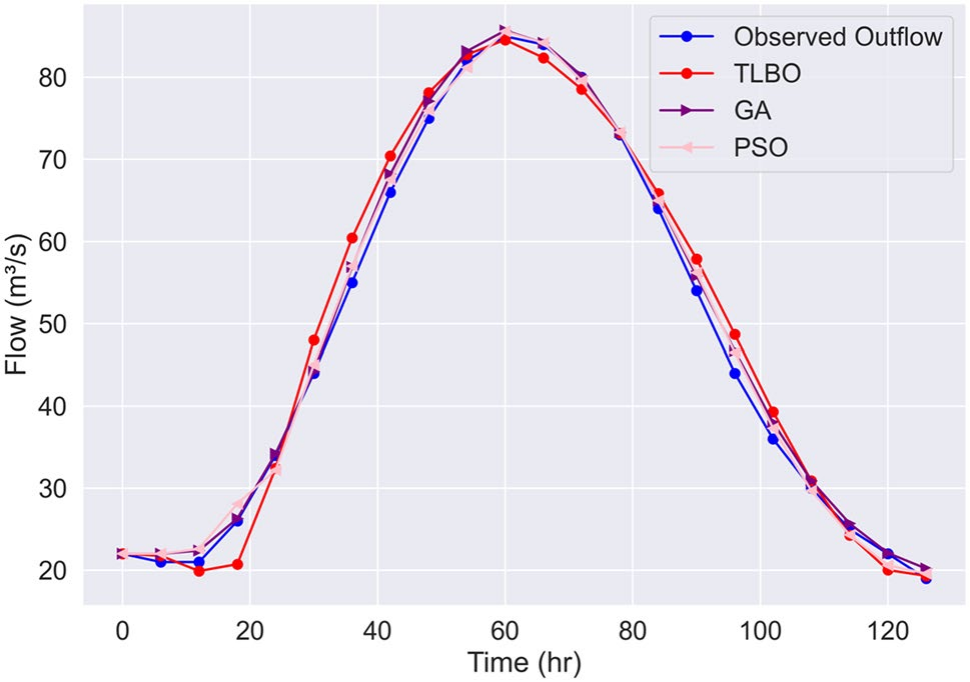
Comparison of TLBO, GA^18^, and PSO^22^ performance accuracy simulating the observed outflow timeseries in the Wilson benchmark.

For comparison purposes, the results from coupling GA^18^ and PSO^22^ to three-parameter Muskingum flood routing for Wilson example with the same objective function have been extracted and presented in Fig. 6. The sensitivity analysis has been implemented in the application of both GA and PSO to increase the accuracy of optimization-simulation Muskingum flood routing^18,22^ which is computationally expensive and time-consuming, additionally the optimization perform sensitive to the algorithmic parameters to reach the global optima. The *SSD* value for fine-tuned GA and PSO are 23.0 and 36.9, respectively. In addition, deviation of peak flood (*DPO*) and deviation of peak time (*DPOT*) have been considered for comparison. The results show that all three optimization algorithms are equally efficient in terms of *DPOT* (*DPOT*_TLBO_ = *DPOT*_GA_ = *DPOT*_PSO_ = 0), while TLBO outperforms the GA and PSO in terms of *DPO*; *DPO*_TLBO_ = 0.42, *DPO*_GA_ = 0.70, and *DPO*_PSO_ = 0.60. This demonstrates the TLBO’s capability to estimate the peak flood value which is a critical value in flood routing accuracy. It should be noted that the performances of the GA and PSO were obtained after parameter calibration, while TLBO reached the solutions without algorithmic parameter tuning.

## Concluding remarks

There are many evolutionary optimization algorithms such as the Genetic Algorithm (GA) and Particle Swarm Optimization (PSO) among well-known ones, and Cat Swarm Optimization (CSO) and Flower Pollination Algorithm (FPA) among recently developed ones. These algorithms calculate near global optimal solutions for complex problems. Yet, their performance relies on the pre-calibration of algorithmic parameters, for there is no deterministic method for their assignment. The specification of evolutionary algorithmic parameters is commonly guided by experienced gained with similar optimization problems, if available. This paper implemented TLBO to estimate the three parameters of the nonlinear Muskingum models. TLBO was coupled with a nonlinear Muskingum flood routing model to make optimal predictions of outflow hydrographs by means of $K$,$\chi$, and *m* calibration, which is the major challenge in Muskingum application for flood routing purposes. The coupling of TLBO with Muskingum routing bypassed the need for specific algorithmic optimization parameters, which are not required in TLBO. The results of *NSE* index (0.99 and 0.94 for the Wilson and Wye River examples, respectively) demonstrate the excellent performance of TLBO for estimating the parameter values rapidly without requiring fine-tuning of optimizing algorithmic parameters. The excellent performance of TLBO with the 3-parameter Muskingum model makes the TLBO a suitable candidate to tackle the 4-parameter Muskingum routing problem in future works, which may lead to even better accuracy of Muskingum flood routing.

## Data Availability

The data that support the findings of this study are available from the corresponding author upon reasonable request.
